# A Multi-Site Phase I Trial of Veliparib with Standard Radiation and Temozolomide in Patients with Newly Diagnosed Glioblastoma Multiforme (GBM)

**DOI:** 10.21203/rs.3.rs-3466927/v1

**Published:** 2023-10-24

**Authors:** Lawrence Kleinberg, Xiaobu Ye, Jeff Supko, Glenn H.J. Stevens, Hui-Kuo Shu, Tom Mikkelsen, Frank Lieberman, Glenn Lesser, Emerson Lee, Stuart Grossman

**Affiliations:** Johns Hopkins University; Johns Hopkins School of Medicine; Harvard medical School; Cleveland Clinic; Emory University; Henry Ford Health; University of Pittsburgh School of Medicine; Wake Forest School of Medicine; Johns Hopkins School of Medicine; Johns Hopkins School of Medicine

## Abstract

**Purpose:**

A multi-site Phase I trial was conducted to determine the safety, maximum tolerated dose, and pharmacokinetics (PK) of Veliparib, a Poly (ADP-ribose) polymerase [PARP] enzyme inhibitor, when administered with temozolomide (TMZ) alone and then with temozolomide and radiation (RT) in patients with newly diagnosed glioblastoma.

**Methods:**

Given the potential for myelosuppression when a PARP inhibitor is combined with chemotherapy, the first 6 patients accrued were given Veliparib 10 mg bid and TMZ 75 mg/m2/d daily for six weeks. If this was well tolerated, the same doses of Veliparib and TMZ would be tested along with standard radiation with plans to dose escalate the Veliparib in subsequent patient cohorts. Once a maximal tolerated dose was determined, a 78 patient phase II study was planned. Peripheral blood pharmacokinetics were assessed.

**Results:**

Twenty-four patients were enrolled. In the first 6 patients who received 6 weeks of TMZ with Veliparib only one dose limiting toxicity (DLT) occurred. The next 12 patients received 6 weeks of RT + TMZ + veliparib and 4/12 (33%) had dose limiting hematologic toxicities. As a result, Veliparib was reduced by 50% to 10 mg BID every other week, but again 3/3 patients had dose limiting hematologic toxicities. The trial was then terminated. The mean clearance (± SD) CL/F of Veliparib for the initial dose (27.0 ± 9.0 L/h, n = 16) and at steady-state for 10 mg BID (23.5 ± 10.4 L/h, n = 18) were similar. Accumulation for BID dosing was 56% (± 33%).

**Conclusions:**

Although Veliparib 10 mg BID administered with TMZ 75 mg/m2 for six weeks was well tolerated, when this regimen was combined with standard partial brain irradiation it was severely myelosuppressive even when the dose was reduced by 50%. This study again highlights the potential of localized cranial radiotherapy to significantly increase hematologic toxicity of marginally myelosuppressive systemic therapies.

## Introduction

There is evidence that Poly (ADP-ribose) polymerase [PARP] enzyme inhibitors may increase the effectiveness of both temozolomide and radiotherapy which are critical in the care of patients with newly diagnosed glioblastoma. It is postulated that PARP inhibitors disrupt DNA damage repair through a number of DNA repair pathways via poly (ADP-ribosyl)ation (PARylation) of histones and DNA repair enzymes.[1–7] The clinical effectiveness of temozolomide, a DNA alkylating agent, is the result of O6-MeG methylation, especially in patients (30%) with methylation of the gene encoding the relevant repair enzyme O^6^-methylguanine-DNA methyltransferase. The more common temozolomide induced methylation at N3 of methyladenine and N7 of methylguanine is efficiently repaired by the base excision repair pathway.[8–13] However, the inhibition of base excision repair by PARP inhibition potentially increases anti-neoplastic effect by decreasing repair at these locations as well.[14] In addition, PARP inhibition may enhance the biologic effectiveness of radiotherapy primarily through impact on single strand break repair, resulting in increased lethal double strand breaks in the context of radiotherapy. [3, 15, 16]

Veliparib is an orally administered small molecule and potent inhibitor of PARP1 and PARP2 that had been well-tolerated with good bioavailability in a preclinical [17]and a phase 0 clinical trial [18]. Veliparib was shown to cross the blood brain barrier in a rodent model. [17]

This background provided strong scientific justification to explore the potential therapeutic benefit of adding veliparib to standard TMZ and RT in the treatment of patients with newly diagnosed glioblastomas. This study was proposed by the NCI funded Adult Brain Tumor Consortium (ABTC) and approved by the Cancer Therapy Evaluation Program (CTEP) of the National Cancer institute. The protocol proposed a phase I component to demonstrate the safety of administering veliparib daily along with six continuous weeks of temozolomide and then determine the maximal tolerated dose and pharmacokinetics when veliparib was added to concurrent standard radiotherapy and temozolomide. The phase II portion of this protocol was designed to estimate survival with this therapy but was not initiated given the findings from the phase I study.

## Methods

### Objective/Outcomes

The primary objective of this phase 1, dose-finding trial was to determine the maximum tolerated dose of veliparib in patients with newly diagnosed glioblastoma when given daily along with standard radiotherapy and temozolomide. Given the potential for excess myelosuppression, a six patient safety cohort was enrolled to first confirm tolerability of administering six weeks of daily veliparib and temozolomide. If this was well tolerated, the second part of the phase I study would be initiated to determine the maximally tolerated dose of veliparib daily when it is combined with concurrent radiation and temozolomide. Secondary objectives were to assess and describe the plasma pharmacokinetics of veliparib and to describe the toxicity. The primary objective for the planned phase 2 was to estimate overall survival.

### Eligibility Criteria

Eligible patients were ≥ 18 years of age with newly diagnosed, histologically-confirmed supratentorial grade IV astrocytoma (GBM), Karnofosky performance status (KPS) ≥ 60%, absolute neutrophil count > 1,500/μL, platelet count > 100,000/μL, hemoglobin ≥ 9.0 g/dL, serum creatinine ≤ 2.0 mg/dL or creatinine clearance ≥ 60 mL/min, total bilirubin ≤ 1.5 mg/dl, and serum transaminases ≤ 2.5-times above the upper limits of institutional normal. Patients in the initial safety cohort of this study were required to have completed standard concurrent RT/TMZ (within 28–42 days of study treatment) including 90% of planned doses of temozolomide without grade ≥ 3 toxicities. Exclusion criteria included pregnancy/breast-feeding; other antineoplastic therapies, life expectancy < 3 months, uncontrolled seizure disorder, inability to swallow/retain oral medication, concurrent cytochrome-P450-inducing anticonvulsants, and comorbidities jeopardizing the ability to receive treatment with reasonable safety.

All subjects provided written informed consent to participate in the trial. The study was reviewed and approved by CTEP and the institutional review boards at each participating ABTC institution.

### Study Design

This was an open-label, multi-center, phase 1 study. The maximum tolerated dose (MTD) was a dose producing a dose-limiting-toxicity (DLT) rate at 33% or less. A modified 3 + 3 design with 6 patients per dose cohort was used due to 10 weeks long safety evaluation period that potentially 40–50% patient might drop-out study for early disease progression.

The initial safety group of up to 6 patients included patients who had recently completed standard concurrent RT/TMZ) were then treated with one cycle of low-dose veliparib 10 mg BID along with 6 weeks of once-a day (QD) temozolomide 75 mg/m2/d (no RT). Once this one cycle was complete, patients were permitted to transition to monthly cycles of standard adjuvant temozolomide 150–200 mg/m2/d for five consecutive days each month.

If safety was confirmed, then the phase I component including radiotherapy would proceed enrolling cohorts of 6 patients to pre-specified escalating dose levels of daily veliparib, ranging from 10 mg BID to 100 mg twice-daily BID daily along with TMZ (75 mg/m^2^/d) for 6 weeks in conjunction with standard radiation 60 Gy in 30 fractions.The protocol specified that if excessive toxicity occurred, de-escalation could be introduced by joint decision of study PI, sponsor, and ABTC leadership to a choice of veliparib 10 mg daily, 10 mg every other day or 10 mg BID 1 week on/1 week off for the 6-week period.

Patients received a single dose of veliparib one day prior to starting combination therapy with TMZ or RT/TMZ for PK testing after the first single dose as described below. Patients without disease progression, as evaluated by MRI 10-weeks after the initiation of veliparib therapy, received up to four maintenance cycles of the same dose of veliparib in the initial safety cohort or six cycles in phase I veliparib/RT/TMZ cohort. Veliparib was to be given BID for 7 days and TMZ 150–200 mg/m^2^ QD for 5 days, repeated every 28 days, with an imaging evaluation after completing every second maintenance cycle.

All subjects were followed until death, and survival from the date of study enrollment was reported to evaluate for any unexpected adverse results.

A single arm phase II efficacy test at the MTD was planned to include 78 patients with 80% power to detect an observed hazard ratio for death of 0.75 (0.45–0.6) at an alpha level of 0.1 (one-sided) when compared with the reference hazard ratio of 0.6 observed in the phase III trial of RT/TMZ reported by Stupp [19]

### Dose-Limiting Toxicities (DLTs)

DLTs were events considered possible or probably related to veliparib alone, veliparibib/TMZ or Veliparib/RT per CTCAE v.3.0 including: ≥ grade 3 nonhematological toxicities not resolving with anticonvulsants or steroid treatment excluding grade 3 nausea and dermatitis. Hematologic toxicity was considered dose limiting if there was 1) ANC ≤ 500/mm^3^; 2) Platelets ≤ 25,000/mm^3^; 3) Grade 3 toxicity that prevents administration of > 75% of the planned temozolomide doses for the first cycle; 4) interruption of treatment for > 14 days. All subjects were monitored for safety throughout the trial.

### Pharmacokinetics

Plasma samples were collected to define plasma concentration-time profiles. Samples were collected shortly before dosing and at 0.25, 0.5, 1, 2, 3, 4, 6, 8, and 24 hours after the first dose of six weeks of TMZ and again after achieving steady-state conditions on any day of week 3 of the six weeks (omitting the 24 hour specimen). Samples were analyzed by liquid chromatography-mass spectrometry (LC-MS)/mass spectrometry (MS), Agilent 410B Triple Quadrupole LC/MS system (Agilent Technologies, Inc., Santa Clara, CA).

The plasma concentration-time data for veliparib was analyzed by noncompartmental methods using model 200 for extravascular input in WinNonlin Professional version 5.0.1. Samples with concentrations below the LLQ of the assay were excluded. The maximum concentration of the drug achieved in plasma (Cmax) and the time that it occurred (tmax) were based upon the observed values following a given dose. Area under the plasma concentration-time curves for 8 h (AUC8) and 24 h (AUC24) after dosing were estimated using the log- linear trapezoidal algorithm. Routines provided in the Data Analysis ToolPak of Microsoft Excel 2003 (11.8231.8221) SP3, Professional Edition (Redmond, WA), were used for the descriptive statistics and statistical tests. Arithmetic averages and standard deviations were calculated for the time of the maximum drug concentration in plasma (tmax). Geometric means were calculated for all other pharmacokinetic variables. The jackknife technique was used to estimate the standard deviation of geometric means.

## Results

### Patient Characteristics

A total of 24 patients were enrolled in this study from 7 institutions. Six were accrued to the initial safety group, 12 to the concomitant TMZ/RT/veliparib 10mg BID treatment group, and 6 to a de-escalation group receiving 10 mg BID every other week during the RT/TMZ. Patient characteristics are summarized in Table 1. Tumor MGMT methylation and IDH were not routinely available at the time of the study.

### Treatment Received and Toxicity

In the initial safety group of patients treated with daily Veliparib 10 mg BID and TMZ only one of 6 patients (16%) had a DLT (grade 4 thrombocytopenia). This permitted advancement to the second part of this study where daily veliparib 10 mg BID was combined with TMZ 75mg/m^2^/d for 6 weeks along with six 6 weeks of focal cranial irradiation.[19] As 2 of the first 6 patients developed severe hematologic DLT (thrombocytopenia), the protocol was amended to accrue another six patients at this dose level to obtain more information after consultation with ABTC leadership, the sponsor, the study PI and approval by the NCI Cancer Therapy Evaluation Program (CTEP). Of the 12 patients treated at this dose, 4 patients (33%) had severe myelosuppression (3 with platelets <25,000/mm^3,^ and one with neutropenia/thrombocytopenia/anemia). After further consultations with the NCI, the company, and the ABTC, the trial principal investigator decided to reduce the veliparib by 50% to administer 10 mg BID of veliparib every other week of the treatment course (weeks 1,3,5). This de-escalation regimen resulted in 3 of 6 subjects (50%) experiencing DLT, 2 thrombocytopenia and 1 neutropenia. The hematologic toxicity with this regimen was judged high enough that the trial was terminated due to the unacceptable DLT rate at the lowest veliparib dose level of interest. A full accounting of the DLT events are presented in Table 2.

### Pharmacokinetic data

A total of 334 plasma samples from 18 patients were processed. Pharmacokinetic parameters for the initial dose were estimated for 16 of the 24 patients. Pharmacokinetic parameters could not be estimated for 8 patients because the sample at 24 hours after dosing was either not collected (1 patient) or collected after the next dose was taken (7 patients). Steady-state pharmacokinetic parameters were estimated for a dose given on week 3 for 18 patients who received veliparib on a continuous basis for six weeks.

**Figure 1:**
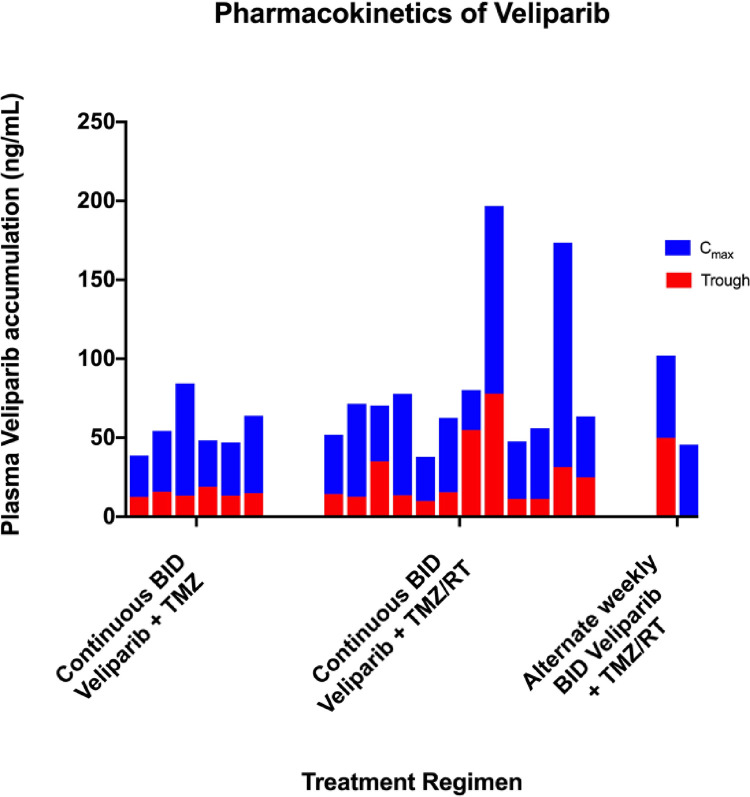
Steady state peak and tough veliparib concentration for each patients getting concurrent veliparib and temoazolomide (6 pts) ,Veliparib/ TMZ/RT (12 pts) ; and every other week veliparib TMZ/RT (samples available for 2 of 6 patients).

The pharmacokinetic parameters were similar for single dose and steady state measurements. (Table 3) Accumulation for BID dosing was 56 ng/mL ± 33%. The steady state peak and trough concentrations for each patient are shown in figure 1.

### Survival

Survival was not a primary outcome for this phase I study. However, the median survival of patients on this study was 13 months (95%CI: 8–16 months) and the survival at 2 years was 25%.

## Discussion

PARP is a nuclear enzyme that recognizes and is activated by DNA damage, catalyzing DNA repair pathways for both single-stranded and double-stranded DNA breaks via poly-ADP-ribosylation of many relevant nuclear target proteins, including histones and DNA repair enzymes.[3, 7, 20, 21] Preclinical studies suggest additional therapeutic benefit to the co-administration of a PARP inhibitor and antineoplastic agents which damage DNA, such as radiotherapy and alkylating agents including TMZ. [1–3, 8, 9, 12, 13, 15, 16, 22]Hence, ABTC initiated a single-arm multi-institutional phase I study to determine the maximal tolerated dose of the PARP inhibitor veliparib administered along with standard postoperative RT/TMZ in the initial therapy of GBM. A preplanned phase II study of the maximal tolerated dose to measure therapeutic outcome did not proceed after a collective decision that even with very low, and likely ineffective, dosing of veliparib a safe dose could not be identified. These discussions involved ABTC investigators, the Cancer Therapy Evaluation Program (CTEP) at NCI, and the pharmaceutical sponsor.

Although randomized controlled trials in other malignant diseases have also demonstrated that PARP inhibition is often associated with some increase in myelosuppression, these studies have confirmed that PARP inhibitors often remain tolerable when administered along with cytotoxic myelosuppressive chemotherapy such as topotecan, gemcitabine, platinum agents, taxanes, cyclophosphamide, and temozolomide.[23–31] A meta-analysis of randomized trials of PARP inhibitor alone or concurrent with systemic chemotherapy demonstrating significantly increased risk of high-grade anemia (RR, 3.06; 95% CI, 2.11–4.43; p < 0.00001), neutropenia (RR, 1.66; 95% CI, 1.33–2.07; p < 0.00001) and thrombocytopenia (RR, 2.76; 95% CI, 1.83–4.16; p < 0.00001).[31] The hematologic effect is hypothesized to be in part consequent to an effect on hematopoiesis consequent to cross-inhibition of PARP-2 which appears to impact hematopoietic progenitor cell survival and capacity for proliferation.[32]

Clinical studies including randomized phase II and III studies using higher daily doses of veliparib have since been published confirming our safety finding that veliparib can be administered to patients with glioblastoma in conjunction with adjuvant TMZ (without concurrent radiotherapy) after completion of standard radiotherapy and also that it is safe along with cranial radiotherapy alone. However, these studies have not provided significant increases in survival. NRG Oncology Group tested a combination regimen of TMZ/veliparib 40 mg BID, dosed according to two randomized schedules (5 vs. 21 days), both alongside TMZ (75mg/m^2^ days 1–5 of 28 day cycle), in the treatment of recurrent TMZ-resistant GBM in patients who had received standard concurrent RT/TMZ in the initial therapy of the disease. [33] The VERTU study of veliparib in the treatment of MGMT-unmethylated GBM patients implemented 200 mg veliparib BID with standard RT alone (without TMZ) followed by concurrent administration of 40 mg veliparib BID on only days 1–7 of each 5 day cycle of standard adjuvant TMZ.[34] Recently presented results of Alliance Trial A071102 (NCT02152982) with randomization of patients with MGMT methylation between standard adjuvant TMZ and veliparib 40 mg BID each day of standard TMZ (days 1–5) after completion of concurrent RT/TMZ also indicated increased but acceptable hematologic toxicity (neutropenia, thrombocytopenia, and anemia) without survival benefit.[35] Moreover, a phase III trial evaluated Veliparib 40 mg BID or 200 mg BID compared with placebo added to whole brain radiotherapy for brain metastasis demonstrated no increase in adverse events. [36]

The demonstrated tolerability of veliparib along with temozolomide alone in this and other studies as well as PK data in the patient dosing groups both suggest that drug accumulation with daily dosing over six weeks is not the mechanism of the observed hematologic toxicity with Veliparib/RT/TMZ. Rigorous pharmacokinetic measurement did not demonstrate alteration of veliparib clearance or drug accumulation over time when co-administered with temozolomide as a potential explanation for increased myelosuppression as clearance, peak, and trough doses remained similar throughout the course of treatment. Other trials have similarly demonstrated no effect of TMZ co-administration on the clearance of veliparib.[37–41] Although temozolomide concentrations were not measured in our study, one trial has confirmed that veliparib coadministration does not affect TMZ plasma exposures.[42]

As the 10 mg BID dose of veliparib with TMZ alone was tolerable and drug accumulation was not observed, we consider it likely that the additional observed toxicity is related to the systemic myelosuppressive effects of localized radiotherapy. The importance of considering the effects of cranial radiotherapy on the significant proportion of blood that flows through the brain during repeated treatment sessions was previously noted when there was worse immunosuppression and thrombocytopenia when radiotherapy was added to BCNU and Cisplatin for glioblastoma [43]. The enhanced hematologic toxicity which may occur when radiation is combined with systemic drugs appears to be mediated through direct effect circulating blood cells .[44–49] The toxicity observed in this trial when adding cranial RT to Veliparib/TMZ again highlights the potential that combining drug regimens with localized cranial radiotherapy may not only result in neurotoxicity but may unexpectedly increase systemic adverse events.

In retrospect, our trial design had several limitations that emphasize important considerations that should be kept in mind while studying sensitizing agents in this disease. The initial safety arm that was designed to demonstrate safety of veliparib along with TMZ (without concurrent radiotherapy) was confined to the select population of patients who had already tolerated concurrent radiotherapy and TMZ without significant myelosuppression. This study also illustrates the need for flexibility in dosing step changes based on unexpected findings with novel multi-agent, multi-modality combination therapies. In this study, the choice of several veliparib dose de-escalation strategies was written into the protocol with decision to be determined by the PI, sponsor, and ABTC based on the observed toxicities and pharmacokinetics. Still, the protocol required amendment to enroll additional patients when 33% thrombocytopenia was observed the first cohort of 6 patients to learn more to guide the decision about dose de-escalation. Temozolomide dose de-escalation was not included in our trial design as there was concern that this could be inappropriate in a trial including a novel agent of unknown effectiveness given concern that concentration of temozolomide behind the blood brain barrier might become too low for therapeutic benefit. However, development of combination drug regimen including temozolomide may ultimately require a temozolomide dose reduction and could be considered in the trial design of select promising new agents where there is meaningful pre-existing evidence of clinical effectiveness.

## Conclusion

Daily Veliparib 10 mg BID can be safely administered with TMZ 75 mg/m2 for six week. However, when these agents are administered with six weeks of standard radiation, a tolerable dose of veliparib could not be found even with 50% dose reductions. Veliparib pharmacokinetics demonstrated peak and trough concentrations remains stable throughout six weeks of daily dosing of veliparib 20 mg BID when administered concurrently with daily temozolomide. This trial highlights the importance of recognizing that radiation may enhance systemic toxicity of drug therapy in trial designs for treatment of newly diagnosed glioblastoma.

## Figures and Tables

**Table 1.** Subject demographic characteristics

	Dosing Schedule			

Variable	Veliparib 10 mg BID + TMZ, *n (%)*	Veliparib 10 mg BID + TMZ + RT, *n (%)*	Veliparib 10 mg BID, alternate weeks, + TMZ + RT, *n (%)*	Total, *n (%)*

**Sex**				

Female	1 (16.7)	5 (41.7)	2 (33.3)	8 (33.3)

Male	5 (83.3)	7 (58.3)	4 (66.7)	16 (66.7)

**Race**				

White	6 (100)	12 (100)	6 (100)	24 (100)

Black	0	0	0	0

American Indian/Alaska Native	0	0	0	0

Asian	0	0	0	0

Other	0	0	0	0

**Ethnicity**				

Hispanic	0	0	0	0

Not Hispanic,	6 (100)	12 (100)	6 (100)	24 (100)

**Age (years)**				

≤25	0	0	0	0

26-65	6 (100)	9 (75)	4 (66.7)	19 (79.2)

66-75	0	2 (16.7)	2 (33.3)	4 (16.7)

>75	0	1 (8.3)	0	1 (4.2)

**Table 2.** Dose limiting adverse Events Possibly Related to Veliparib alone or of the combination with TMZ or TMZ/RT

	Dosing Schedule			

Adverse event, MedDRA 14.1	Veliparib 10 mg BID + TMZ, *n (%)*	Veliparib 10 mg BID + TMZ + RT, *n (%)*	Veliparib 10 mg BID, alternate weeks, + TMZ + RT, *n (%)*	Total, *n (%)*

**Any adverse event**	3 (50)	5 (41.7)	3 (50)	11 (45.8)

**Blood and Lymphatic Systemic Disorders**				

Anemia	0	1 (8.3)	0	1 (4.2)

Febrile neutropenia	0	1 (8.3)	0	1 (4.2)

Neutropenia	0	1 (8.3)	1 (16.7)	2 (8.3)

Pancytopenia	0	0	1 (16.7)	1 (4.2)

Thrombocytopenia	1 (16.7)	4 (33.3)	2 (33.3)	7 (29.2)

Subjects with ≥ 1 AE	1 (16.7)	4 (33.3)	3 (50)	9 (37.5)

Abbreviations: NCI CTCAE = National Cancer Institute Common Terminology Criteria for Adverse Events; MedDRA = Medical Dictionary for Regulatory Activities; AE = adverse event.

**Table 3.** Pharmacokintic measurements.

	Single dose 10 mg Veliparib	Steady-state 10 mg BID Veliparib
No. of patients	16	18
C_min_ (ng/mL)	2.5 ± 1.3	18.4 ± 10.4
C_max_ (ng/mL)	50.3 ± 19.0	66.1 ± 28.7
t_1/2,z_ (h)	5.8 ± 0.9	Not applicable
AUC (n·h/mL)	370 ± 126	425 ± 181
CL/F (L/h)	27.0 ± 9.2	23.5 ± 10.4
Accumulation (%)	Not applicable	56+33

Abbreviations: a Cmin, minimum drug concentration in plasma; Cmax, maximum drug concentration i plasma; t1/2, apparent terminal phase half-life; AUC, area under the plasmaconcentration-time curve to infinity (single dose) or for a 12 h dosing interval (steady-state); CL/F, apparent oral clearance per hour.

## Abbreviations

a Cmin: minimum drug concentration in plasma
Cmax: maximum drug concentration I plasma
t1/2: apparent terminal phase half-life
AUC: area under the plasmaconcentration-time curve to infinity (single dose) or for a 12 h dosing interval (steady-state)
CL/F: apparent oral clearance per hour
